# Treatment of Gunshot Fractures of the Mandible

**Published:** 1919

**Authors:** 


***Treatment of Gunshot Fractures of the Mandible.*** By JOHN B. ROBERTS. *Annals of Surgery*, September, 1918.

The shape, situation and function of the lower jaw, and its relation to other facial structures, lend to the vulnerating missile an extraordinary opportunity for serious complicating lesion.

When one recalls the shape and function of the mandible, the real reason for its frequent malformation after union of a fracture is obvious.

It is fortunate for patients who sustain fractures of the mandible that the two maxillae above furnish an immovable anvil against which the teeth of the mandible strike in chewing food. This anvil-like mass of bone and teeth may be utilized by a surgeon as a splint to support and steady the broken lower jaw after its fragments have been so replaced as to reconstruct the dental arch.

The usual fractures occurring in the body of the mandible are not difficult to reduce and keep reduced if both jaws have intact teeth

In comminuted injuries the reduction is not apt to be easy, and its maintenance may be difficult.

After the surgeon has brought the fragments into their position in uncomplicated fractures, the upper and lower teeth should be kept in contact by closing the mouth, and then holding the mandible firmly against the upper jaw by a figure of eight bandage of occiput and chin, or by some similar appliance to prevent the patient opening his mouth. When a simple bandage will not give sufficient firmness, and when its lateral or backward pressure causes overriding of fragments, a moulded splint should be applied to the outside of the skin to constitute a hollow cap fitting the front and lower surfaces of the mandibular region.

If the tendency to displacement is persistent, wiring the fragments together, or fixing one or more teeth of the mandible against those of the upper jaw by wires, carried across from the teeth of the mandible to the teeth of the maxillae, may be a valuable expedient. Sometimes fixation by an intraoral or dental splint becomes necessary. A temporary splint may be made by softening a gutta-percha strip in hot water, moulding it to the crown of the lower teeth so as to overlap the adjacent gums, and hardening it with cold water.

Union of ordinary fracture of the mandible takes place in about five weeks. In many cases apparently likely to give bad position, there is ultimately quite a good result provided that sepsis does not occur, and a fairly good apposition of fragments is maintained during the early stages.

The normal occlusion of the teeth should be reestablished in gunshot fractures as soon as possible, even before there is any general suturing of strips of tissue, if these are greatly lacerated. Unless this is accomplished the fracture displacement will probably become permanent, and reconstruction of the contour of the face very difficult to effect.

Most gunshot fractures of the mandible are open and, therefore, liable to become contaminated, and later infected. The raw surfaces should be cleansed and well painted with tincture of iodine, dicholoramine-T, or other antiseptic, and vulnerating missile and foreign bodies thoroughly removed as soon as possible after injury.

The soft parts may be brought together over the broken bone after the fracture has been reduced. They should not be sutured so close as to interfere with drainage if there is a probability of infection becoming marked.

The drainage will be particularly needed in damaged tissues of the lower facial region and chin. This should be provided for in some cases of fracture of the lower jaw by incision below the inferior margin of its body.

Gunshot fractures of the mandible are so essentially open fractures in most cases that osteomyelitis and other septic complications are common. Necrosis may thus impede union and cause permanent non-union with atrophy of the ends of fragments. Bone transfer by flap from clavicle, or grafting from rib or tibia, may enable the surgeon to reconstruct the mandibular arch; or he may use a graft of costal cartilage for this purpose.

